# The Relative Age Effect, Height and Weight Characteristics among Lower and Upper Secondary School Athletes in Norway and Sweden

**DOI:** 10.3390/sports5040092

**Published:** 2017-12-08

**Authors:** Stig Arve Sæther, Tomas Peterson, Vazjwar Matin

**Affiliations:** Department of Sociology and Political Science, Norwegian University of Science and Technology, NTNU, 7491 Trondheim, Norway; tomas.peterson@mah.se (T.P.); vazjvar.matin@hotmail.com (V.M.)

**Keywords:** the relative age effect, upper secondary school, lower secondary school, weight, height

## Abstract

The relative age effect (RAE) has been found among youth elite athletes within a range of sports. However, the RAE has been studied to a lesser degree among youth non-elite athletes, and even less among school pupils attending sport specialisation programmes (SSPs). The aim of the present study was to investigate RAE, height, and weight, and compare Swedish lower secondary school and Norwegian upper secondary school pupils. Study 1 includes 156 lower secondary school athletes (95 boys and 61 girls) following an SSP in Sweden, while study 2 includes 111 upper secondary school athletes (81 boys and 30 girls) from two Norwegian schools. The RAE was found in both male groups, but only in Swedish girls. Furthermore, the relationship between birth month, height, and weight was found to be non-significant. These results indicate a vital RAE effect among youth non-elite athletes attending SSPs in both lower and upper secondary schools.

## 1. Introduction

The value of children’s and youth sport is paradoxical. On the one hand, research shows that sport and physical activity during childhood and adolescence (5–18 years) has important implications for an individual’s physical and mental health [1]. Physical activity promotes both physical development and motor development, and prevents illness. People expect former student-athletes to display significantly more leadership, self-confidence, and self-respect than those who were active outside of sports—such as being in the band or on the yearbook staff. Men who participated in varsity-level high school sports an average of 60 years earlier appeared to demonstrate higher levels of leadership and enjoyed higher-status careers, and also exhibited more prosocial behaviour than non-athletes [2]. Thus, it seems imperative to encourage young people, both during school and leisure time, to be physically active. On the other hand, research demonstrates that sporting activities offered by schools and sport clubs may have negative consequences. A narrow focus on results and performance may result in the exclusion of some children and young people, who consequently become uninterested in sport and physical activity [3]. In addition, sport activities may be harmful socially, psychologically, or physiologically for young people [4].

In recent years, many lower secondary schools in Sweden have specialised in physical activity and sports. This trend has been both acclaimed and criticised [5,6], and the impact of these sports profiles is unclear. We do not know if these pupils become elite athletes in adulthood. Furthermore, it is not known to what extent the pupils in these schools even take part in competitive or recreational sports in adulthood compared to their peers in ordinary schools. The same trend is also seen in Norway, but to a much lesser extent than in Sweden.

However, sport specialisation programmes (SSPs) and elite sport specialisation programmes (ESSPs) in upper secondary schools are much more common both in Norway and Sweden. These types of schools have been regarded as an essential part of a young athlete’s development process; in particular, football and skiing clubs have made a close relationship in order to create a holistic development environment, since these young people are both players and pupils. Dilemmas in this regard concern issues such as training load, burnout, and stress regarding the pupil-athlete dilemma these young people are facing.

One of the outcomes of the way in which both school sports and voluntary sports are organised to produce elite athletes is the unintended use of differences in physical maturity within a group of selected athletes [7], described as the relative age effect (RAE). The existence of the RAE is now a widely recognised effect of selection systems within competitive children’s and youth sport, across sports and countries all over the world [8]. However, RAE is complex and varies across sports and genders. Romann and Fuchslocher [9] analysed birth date distributions of female athletes in Swiss Youth sports and the subgroup of the National Talent Development Program (TDP) in individual sports, where comparisons showed significant RAEs in the distribution of athletes’ birth dates in alpine skiing, tennis, athletics, fencing, and snowboarding. Significant “reverse” RAEs with an overrepresentation of athletes at the end of the year were found in table tennis. In the TDP, significant RAEs were found in alpine skiing and tennis. No RAEs were detected in athletics. In table tennis, fencing, and snowboarding, “reverse” RAEs were found. Delorme and Raspaud [10] observed a significant RAE in all youth categories of French basketball players, aged from 7 to 17 years, where the effects were even more pronounced for girls than boys. Lately, evidence has shown a lack of RAE among female athletes [8], which questions whether the effect is as prevalent among female athletes as male. The relationship between body mass, height, and the RAE has been found to be present among males, but to a lesser extent among female athletes [10,11].

In addition, while RAE has been well-established in junior hockey and other professional sports [12], the long-term impact of this phenomenon is not well understood. In a study of NHL players from the 2008–2009 season to the 2015–2016 season, Fumarco et al. [13] documented a RAE reversal—players born in the last quarter of the year score more and command higher salaries than those born in the first quarter of the year. According to McCarty et al. [14], the initial bias of RAE appears to not be systemic in some talent development systems. They report an investigation into the initial identification, selection, and conversion of academy players from professional Rugby Union and Cricket at national level. Data demonstrated a reversal of RAE advantage, whereby relatively young players from both sports were less likely to be selected into their respective national academy systems but were more likely to transition into senior national squads. Different patterns have been noted in different sports.

RAE in sports has often been used as a model for discussions about RAE in Education or other fields. Kniffin and Hanks [15] examined the influence of RAE upon specific factors related to earning a PhD: age at degree, time to degree, and salary upon completion. They found no significant influence of RAE on the age of people earning the PhD and no influence on post-graduate salary. To the extent that earning the PhD is considered an outstanding achievement, their findings support the view that redshirting is unnecessary and costly. Du et al. [16] found that the number of corporate CEOs born in June and July is disproportionately small relative to the number of CEOs born in other months. This is consistent with the RAE due to school admissions grouping together children with age differences up to one year, with children born in June and July disadvantaged throughout life by being younger than their classmates born in other months. The results suggest that the relative-age effect has a long-lasting influence on career success.

Especially within football, this effect has been studied thoroughly, mainly among male national youth team soccer players; the effect has been shown to be strongest in European countries such as the United Kingdom, Sweden, and Belgium [17]. Williams [18] found a strong effect among U17 world cup players, while Helsen and his colleagues [19] found the RAE among ten European U15–U18 national teams. Moreover, studies in Sweden [20], Norway [21], Spain [22], and France [23] have also detected an RAE among youth players. Even though there are few studies involving female players, Delorme and colleagues [24] found an RAE among French players; these results were also related to a higher drop-out among players born in the two last quarters of the year.

However, there are studies confirming that RAE can be connected to performance in physical education among pupils in a British secondary school [25], British lower secondary schools [26], Norwegian lower secondary schools [27], British upper secondary schools [28], and Norwegian upper secondary schools [29].

The aim of the present study was to investigate the RAE, height, and weight characteristics, and compare Swedish lower secondary school attending an SSP and Norwegian upper secondary school pupils attending an SSP or ESSP.

## 2. Materials and Methods

### 2.1. Study 1

#### 2.1.1. The MYSP Project

The Malmö Youth Sport Program (MYSP) started in 2012, and is a longitudinal study following boys and girls at a local lower secondary school, and is a continuation and development of the “Bunkeflo project” [30,31] which, for nine years, examined the relationship between children’s health, academic achievement, and engagement in sport and physical activity at school. The overall aim of the MYSP study is to study lower secondary schools which offer SSP and see how they contribute to fostering elite athletes and, secondly, to suggest whether these types of schools contribute to pupils taking part more in competitive or recreational sports in adulthood compared to their peers in schools not offering these programmes. Participants comprised pupils born in 2000 and 2001 attending the school.

#### 2.1.2. Sample and Data Collection

The participants included 61 girls and 95 boys representing one Swedish lower secondary school offering an SSP. The players had a mean age of 13.49 years (SD 0.32) and were born in 2000 and 2001; a total of 12 different sports were represented.

#### 2.1.3. Procedure and Data Analyses

The data were collected using a questionnaire among two different age cohorts. Before answering the questionnaire, all participants were informed about the purpose of the study, that their participation was voluntary, that the survey was anonymous, and that all information would be treated confidentially. All subjects gave their informed consent for inclusion before they participated in the study. The study was conducted in accordance with the Declaration of Helsinki, and the protocol was approved by the Ethics Committee of Malmö University. The pupils’ month of birth was categorised into quarters reflecting the Swedish soccer year. The first quarter includes January, February, and March, and the fourth quarter includes October, November, and December. The results are presented with basic descriptive statistics, such as frequency counts and percentages. Chi-square tests were performed to compare differences between the observed and expected birth rate distribution across the four quarters of the Swedish soccer year. All analyses were conducted using SPSS version 22.0 (IBM Corporation, Somers, NY, USA).

### 2.2. Study 2

#### 2.2.1. The Upper Secondary School Project

The upper secondary school study investigated football players attending one SSP and one ESSP in terms of the hallmarks these players identified in 2016. This cross-sectional study investigated the pupils’ assessment of their own skills and asked them to rank different skills from most to least important as criteria for talent identification in football.

#### 2.2.2. Sample and Data Collection

The participants included 30 girls and 81 boys representing two Norwegian upper secondary schools offering an SSP or ESSP. The players had a mean age of 17.47 years (SD 0.77), were born between 1997 and 1999, and included only football players.

#### 2.2.3. Procedure and Data Analyses

The data were collected using a questionnaire among pupils from two upper secondary schools. Before answering the questionnaire, all participants were informed about the purpose of the study, that their participation was voluntary, that the survey was anonymous and that all information would be treated confidentially. All subjects gave their informed consent for inclusion before they participated in the study. The study was conducted in accordance with the Declaration of Helsinki, and the protocol was approved by Norwegian Social Science Data Services (Project identification code: 47571).

Each player’s month of birth was categorised into quarters reflecting the Norwegian soccer year. The first quarter includes January, February, and March, and the fourth quarter includes October, November, and December. The results are presented with basic descriptive statistics, such as frequency counts and percentages. Chi-square tests were performed to compare differences between the observed and expected birth rate distribution across the four quarters of the Norwegian soccer year. All analyses were conducted using SPSS version 22.0. The independent *t*-test was used to identify significant differences between the Norwegian SSP and ESSP schools. The significance level (alpha) was set to 0.05.

## 3. Results

### 3.1. Birth Month

The results showing an RAE effect indicated that 63% of the pupils were born in the first six months of the year, and only 14% were born in the last three months (Table 1). Furthermore, the results showed similarities among the Norwegian and Swedish pupils, with only non-significant differences.

Comparing boys and girls, the results showed that there were more pupils born in the first three months among the Norwegian compared to the Swedish boys, but the differences were small, and even smaller when comparing those born in the first six months (Table 2). Among the girls, there were more Swedish pupils born in the first three months, but the distribution was similar when we included the first six months. Comparing the pupils born in the last three months of the year, there were more pupils born in these months among the Swedish girls, and more pupils born in these months among the Norwegian boys. Analysed separately for gender, there was no significant difference between boys and girls.

Comparing the four age groups in the present studies, the results showed similarities among the 13 year-olds, the 17 year-olds, and the 18 year-olds (Table 3). Approximately one out of three was born in the first three months, two out of three were born in the first six months, and only approximately one out of ten was born in the last three months. The results from the 19 year-olds are quite different, since one out of five was born in the first three months and three out of ten were born in the last three months; this was the only age group with no significant differences among the quarters. Even so, also among this group, close to two out of three were born in the first six months.

### 3.2. Height and Weight

The results showed that the height of the Norwegian athletes was mainly between 170–190 cm; that of the Swedish athletes was between 150–170 cm (Table 4). The weight of the Norwegian athletes ranged between 65–79 kg; that of the Swedish athletes was between 45–59 kg (Table 5). These differences could mainly be explained by the different age cohorts in the Norwegian and Swedish samples.

### 3.3. Relationship—RAE, Height, and Weight

The results showed no significant correlations between birth quarter and gender, weight, and height (Table 6). As expected, a relationship between age, height, and weight was shown among both Norwegian and Swedish athletes, and between gender, height, and weight—the boys were significantly taller and heavier than the girls. There was, however, a close-to-significant (*p* = 0.089) correlation between the Swedish athletes’ height and birth month. Among the three age cohorts of the Norwegian players, no significant correlation was found between birth month and height or weight. No significant difference (using the *t*-test) was found between the school types in Norway regarding birth quartile, height, or weight.

## 4. Discussion

The RAE has been widely documented in different sports [11], but especially among male youth elite football players [18,19]. This chronological age grouping of youths—both girls and boys—tends to advantage the players born in the early part of the selection year. This effect has—albeit to a lesser degree—been studied among players at a lower level, among both boys and girls. The relationship between this player characteristic and the presence of this effect among upper secondary school pupils has not been studied, even though studies have been carried out that show that the physical education grade and the RAE is present, both in lower secondary schools [26,27] and upper secondary schools [28,29]. The results from this study showed that the RAE was present among both Swedish and Norwegian school pupils attending SSPs. While we are aware of the limitations of the study, especially with respects to the sample sizes, the results are in line with most findings related to the existence of RAE. The results showed that the RAE was present among both Swedish and Norwegian school pupils attending SSPs. Approximately two out of three athletes were born in the first half of the year, whereas only about 14% were born in the last quarter of the year. These results are perhaps not too surprising, since admission to ESSP schools is very competitive. Even so, this could indicate that the RAE is more of an issue among athletes with a lower level of skills. Furthermore, the RAE was also present at SSP schools, in which admission was less competitive.

The RAE was also found in boys in both Norway and Sweden, but only among the Swedish girls. One reason could be that the sample of Norwegian girls was smaller than the sample of Swedish athletes, and that the Norwegian athletes were football players only, while the Swedish athletes represented a range of different sports. Even so, earlier studies have found the RAE to be more prevalent among male athletes [12]. Furthermore, the RAE was found in all age groups, with the exception of the 19 year-old players. One explanation for these results could be the known disappearance of this effect as the athletes become older and the selection process changes. In most sports, the players regarded as the most talented are characterised by an early birth month, and have the obvious advantages of being older and more mature than the younger athletes [7]. These athletes’ skills are therefore often related both to their physical and psychological age advantage. However, when the youngest athletes progress, many of the advantages disappear. However, the studied sample was not characterised by such a change among the selected athletes, so there probably exist different reasons as to why the RAE was not found among this group of athletes.

Even though one could expect a relationship between birth month, height, and weight, no such relationship was found in the present study. There was, however, one exception: in the Swedish athletes, there was found a close-to-significant (*p* = 0.089) correlation between height and birth month. Analysed separately for gender, there was no significant relationship. The lack of correlations between height, weight, and birth quarter must be considered surprising, since one could expect the differences to occur naturally among boys maturing later. Because earlier studies have not found a relationship between body mass, height, and RAE, the results from the female athletes were less surprising [32].

These results could indicate that much of the explained advantages possessed by athletes born early in the year—often related to the physical advantages of being taller and/or heavier—were not seen in this sample of athletes. Even so, the advantage of being older and thereby more psychologically mature is an advantage independent of the missing physical advantages. Another potential explanation could be that the pupils have already taken advantage of the RAE earlier in the process. These results would indicate that the RAE is also a vital effect among youth non-elite athletes, attending an SSP and ESSP in both lower and upper secondary schools.

## Figures and Tables

**Table 1:** Descriptive data on birth month in the total sample, and the Norwegian and Swedish samples separated.

Quarter	Total Sample		Norwegian Athletes		Swedish Athletes	
	Sample Size	Percent	Sample Size	Percent	Sample Size	Percent
1	90	33.6	36	3.4	54	34.6
2	80	30	36	32.4	44	28.2
3	60	22.5	23	20.8	37	23.7
4	37	13.9	16	14.4	21	13.5
Total	267	100	111	100	156	100
Chi-square	x^2^ = 24.67	*p* = 0.00	x^2^ = 10.69	*p* = 0.01	x^2^ = 14.82	*p* = 0.00

**Table 2:** Descriptive data of birth month in the total sample, and the Norwegian and Swedish samples separated, according to gender.

Quarter	Total		Boys		Girls	
	Boys	Girls	Norway	Sweden	Norway	Sweden
1	32.4	36.3	34.6	30.5	26.7	41
2	35.8	18.7	34.6	36.8	26.7	14.8
3	19.3	28.6	16	22.1	33.3	26.2
4	12.5	16.5	14.8	10.5	13.3	18
*N*	111	156	81	95	30	61
x^2^	25.31	9.17	11.88	10.01	2.53	14.76
*p*	0.00	0.02	0.00	0.00	0.46	0.01

**Table 3:** Descriptive data of birth month in the total sample, according to age.

Quarter	13 years	17 years	18 years	19 years
1	34.6	33.3	38.1	19
2	28.2	35.4	23.8	42.9
3	23.7	18.8	28.6	9.5
4	13.5	12.5	9.5	28.6
*N*	156	48	42	21
x^2^	14.82	7.16	7.14	5.09
*p*	0.00	0.06	0.06	0.16

**Table 4:** Descriptive data of height in the total sample, and the Norwegian and Swedish samples separated, in cm.

Height	Total Sample		Norwegian Athletes		Swedish Athletes	
	Sample Size	Percent	Sample Size	Percent	Sample Size	Percent
140–149	9	6.1	0		9	6.1
150–159	51	35.6	4	3.6	47	32
160–169	76	53.9	10	9	66	44.9
170–179	63	52.6	44	39.7	19	12.9
180–189	52	45.5	46	41.4	6	4.1
190–199	7	6.3	7	6.3		
Total	258	100	111	100	147	100

**Table 5:** Descriptive data of weight on the total sample, and the Norwegian and Swedish samples separated, in kg.

Weight	Total Sample		Norwegian Athletes		Swedish Athletes	
	Sample Size	Percent	Sample Size	Percent	Sample Size	Percent
30–34	1	0.7	0		1	0.7
35–39	13	8.8	0		13	8.8
40–44	21	14.3	0		21	14.3
45–49	39	27.3	0		39	27.3
50–54	27	20.2	6	5.5	21	14.7
55–59	32	23.1	3	2.8	29	20.3
60–64	30	24.9	18	16.5	12	8.4
65–69	28	24.6	23	21.1	5	3.5
70–74	28	24.8	24	22	4	2.8
75–79	25	22.5	23	21.1	2	1.4
80–84	8	7.3	8	7.3		
85–89	4	3.6	4	3.6		
Total	256	100	109	100	147	100

**Table 6:** Correlations between age, gender, birth-month, height, and weight, Norwegian and Swedish samples separated.

Athlete Characteristics	Norwegian Athletes					Swedish Athletes		
	Age	Gender	Quarter	Height	Weight	Gender	Quarter	Height
Age								
Quarter	−0.061	0.061				−0.008		
Height	−0.121	−0.692 **	−0.013			−0.104	−0.141	
Weight	−0.302 **	−0.656 **	−0.024	0.835 **		0.075	−0.128	0.800 **

** Statistically significant, *p* < 0.01.
